# Social–Emotional Learning for students with ADHD: investigating teacher perspectives and practices

**DOI:** 10.3389/fpsyg.2026.1694782

**Published:** 2026-02-24

**Authors:** Ghram A. S. Kushi, Keetam D. F. Alkahtani

**Affiliations:** 1 Doctoral Program at the Department of Special Education, College of Education, King Saud University, Riyadh, Saudi Arabia; 2 Department of Special Education, College of Education, King Saud University, Riyadh, Saudi Arabia

**Keywords:** Attention Deficit/Hyperactivity Disorder (ADHD), mixed-methods, self-assessment, Social–Emotional Learning (SEL), teachers, inclusive education, holistic student development

## Abstract

**Background:**

Social-Emotional Learning (SEL) is a powerful approach for helping students develop essential life skills. For students with Attention-Deficit/Hyperactivity Disorder (ADHD), these skills are particularly vital. The five core competencies of SEL directly address the executive function deficits that often accompany ADHD. While SEL is essential for the holistic and academic success of these students, its effective implementation depends heavily on teachers’ attitudes and beliefs. This study investigates teacher perceptions and practices regarding SEL for students with ADHD and identifies the specific challenges they face.

**Methods:**

The study employed a mixed-methods approach, utilizing a self-reporting quantitative survey supplemented by three qualitative open-ended questions. The survey administered to 144 primary school teachers (91 female, 53 male). Data analysis focused on how perceptions and practices varied across demographic factors, including gender, job title, experience, and academic qualifications.

**Results:**

Teachers’ perceptions and practices of SEL for students with ADHD differ significantly based on gender; however, no significant differences were found regarding job title, years of experience, or academic degree. Integrated findings from both quantitative and qualitative data indicate that while teachers acknowledge the profound value of SEL, they face substantial systemic barriers that hinder full implementation in the classroom.

**Conclusion:**

There is a clear gap between teachers’ pedagogical beliefs and their actual classroom practices. Closing this gap between belief and practice is essential for the holistic development and academic success of students with ADHD. Without dedicated resources and institutional support, the full potential of SEL to positively impact students with ADHD will remain largely unrealized.

## 1 Introduction

A group of researchers, educators, and child advocates gathered in 1994, driven by the belief that all students’ social and emotional needs should be met. This collaboration resulted in the formation of the Collaborative for Academic, Social, and Emotional Learning (CASEL) and the first appearance of the term “Social–Emotional Learning (SEL).” The goal was, and still is, to make high-quality, evidence-based SEL a core part of education at all levels, providing schools and families with the tools needed to raise knowledgeable and skilled generations.

Modern education extends beyond academic knowledge to include character and life skills, preparing students to become productive members of society. Globally, SEL has gained significant attention as a crucial component of student development. Research consistently highlights the efficacy of SEL programs in improving academic outcomes, enhancing positive behaviors, and reducing emotional and behavioral challenges. For example, studies have shown that SEL programs can lead to improved grades, increased student participation, and even higher high school graduation rates (Greenberg, 2023; Li et al., 2023).

School-based Social and Emotional Learning (SEL) programs yield benefits that extend far beyond the classroom. Research, notably the meta-analysis by Durlak et al. (2011), demonstrates that teacher-led classroom interventions across all educational levels significantly enhance social–emotional skills and positive behavior. These comprehensive programs are also linked to a reduction in behavioral distress and a measurable boost in academic performance.

The importance of SEL is further supported by a comprehensive review conducted by Durlak et al. (2022). This review analyzed 12 meta-analyses from 2011 to 2020, covering more than 500 studies from various countries to evaluate the impact of SEL programs on students from preschool through high school. The review found that SEL had a positive impact across the board, leading to improved social and personal skills, better attitudes, and enhanced academic performance. Additionally, it documented a reduction in behavioral problems such as bullying, aggression, emotional distress, and substance abuse. Although the review did not specifically focus on teachers, it highlighted the critical need for both new and experienced teachers to receive training on how to apply educational strategies that promote strong SEL skills.

Therefore, the impact of SEL is universal yet contingent on three factors: quality of delivery, the school’s atmosphere, and teacher involvement. As Durlak et al. (2022) suggest, SEL should be viewed as a foundational element of holistic education that strengthens classroom structure and accessibility without detracting from academic rigor. SEL functions as a vital complement to—not a substitute for—traditional academics, fostering an organized classroom and enhancing learning outcomes for all students.

Teachers are central to the successful implementation of SEL programs in the classroom. When teachers have a positive attitude toward SEL, they are more likely to integrate it into their daily instruction and seek the necessary training. This, in turn, leads to better implementation and greater student engagement, ultimately benefiting students with ADHD by fostering their social, emotional, and academic development.

Given the documented benefits, examining teachers’ opinions and beliefs about SEL is crucial. Teachers with a positive attitude towards SEL are more likely to implement it and seek relevant training and support (Payton et al., 2008; Buchanan et al., 2009; Brackett et al., 2012; Collaborative for Academic, Social, and Emotional Learning (CASEL), 2020; Yoder, 2022; Daldal and Tuyan, 2024). This aligns with the broader educational goal of developing students’ character and life skills beyond academic knowledge, preparing them to be productive community members.

Students with Attention-Deficit/Hyperactivity Disorder (ADHD) often face significant challenges due to impairments in their executive functions. This weakness profoundly affects their ability to perform daily tasks, making it particularly difficult for them to succeed in educational environments (Barkley, 2016). Beyond mere attention-related academic struggles, Barkley (1997) identified these difficulties as the disorder’s foundation. They surface through impulsive actions and social hurdles, ultimately disrupting a student’s broader emotional and behavioral adaptation to the school environment.

Consequently, students with ADHD can greatly benefit from SEL programs. These programs are designed to help all students manage their emotions, build positive relationships, make responsible decisions, and foster desirable behaviors (Greenberg, 2023). Conversely, a lack of social and emotional competence can exacerbate psychological, behavioral, and academic problems (Durlak et al., 2022).

Although these SEL programs are not specifically designed for students with ADHD, the conceptual framework and its core competencies outlined by the CASEL—self-awareness, self-management, social awareness, relationship skills, and responsible decision-making—directly address the primary challenges associated with ADHD and aligns closely with the skills these students need to succeed.

Research from CASEL consistently shows that when students develop these five competencies, they see improved academic performance, better social skills, and reduced behavioral issues (Collaborative for Academic, Social, and Emotional Learning (CASEL), 2023). The educational requirements of students with ADHD intersect directly with Social and Emotional Learning (SEL) competencies. Research by Drigas et al. (2025) suggests that learning environments prioritizing social and emotional growth foster improved emotional regulation, positive social interaction, and increased classroom participation for these students. This is further supported by Bakola et al. (2019), who found that ADHD interventions increasingly focus on self-regulation and interpersonal skills, mirroring the core goals of SEL. Furthermore, integrating these practices into mainstream settings can mitigate operational challenges and promote academic inclusion (Drigas et al., 2025). However, the efficacy of SEL depends less on the availability of tools and more on teacher perceptions; educators’ attitudes and their belief in the value of SEL are primary determinants of its success in the classroom (Bakola and Drigas, 2020; Buchanan et al., 2009; Drigas et al., 2025). Given the direct overlap between SEL skills and the challenges faced by students with ADHD, a positive teacher attitude toward SEL can lead to more effective implementation and greater student engagement (Durlak et al., 2022). This, in turn, can significantly improve social, emotional, and academic outcomes for students with ADHD.

In Saudi Arabia, the government has made significant strides in supporting students with ADHD by including them in general education classrooms. Although there is no formal national policy mandating SEL, its principles are promoted through national initiatives like Vision 2030, which aims to foster supportive learning environments and promote social integration (Government of Saudi Arabia, 2016). Despite these efforts, there is a lack of research on how teachers in Saudi Arabia understand and implement SEL, especially when working with students with disabilities. This gap is particularly important to address, given that the skills targeted by SEL are directly linked to the executive function challenges faced by students with ADHD.

This study aims to fill this critical research gap by exploring how teachers of students with ADHD in Saudi Arabia perceive and implement SEL in inclusive classrooms. It also examines whether their practices vary based on factors such as gender, educational level, teaching specialization, and years of experience. By doing so, the study will provide valuable insights into how SEL can be more effectively integrated into inclusive educational settings.

## 2 Materials and methods

### 2.1 Methodology and design

This study employed a mixed-methods approach, primarily using a quantitative survey to collect data. A web-based, self-reporting survey was selected to reach a large number of participants and gather numerical data on teachers’ perceptions, practices, and challenges related to implementing SEL. To enrich the quantitative data, the survey was supplemented with a qualitative component featuring three open-ended questions. This mixed-methods design, which is common in educational research, provided a deeper understanding of the quantitative findings by allowing participants to offer more detailed insights. The research design was approved by the Ethics Committee of King Saud University (KSU-HR-24-1137). All participants provided informed consent, and their data confidentiality was maintained throughout the study.

### 2.2 Instrument

We developed a questionnaire to be the data collection tool for this study. The questionnaire was adapted from several sources, including publications from the Collaborative for Academic, Social, and Emotional Learning (CASEL), the Social and Emotional Learning Self-Assessment Scale for Teachers (Yoder, 2022), and other relevant existing literature (e.g., Buchanan et al., 2009; Schonert-Reichl, 2017; Kaur and Sharma, 2022). The questionnaire was structured into five distinct sections. It began by gathering demographic data with seven initial questions. The remaining four sections used a five-point Likert scale (strongly agree to strongly disagree) for all closed-ended questions. These sections explored participants’ perceptions of SEL (12 statements), their classroom practices (7 statements), and the challenges they encountered during implementation (7 statements). The final section of the questionnaire included three open-ended questions to gather additional qualitative insights.

### 2.3 Participants

The participants included 144 primary school teachers, who were presumed to have computer and internet access. The majority of the participants were female (*n* = 91, 63.2%) and general education teachers (*n* = 114, 79.2%). The academic backgrounds of the participants varied, but most held a bachelor’s degree or a higher diploma (*n* = 108, 75.0%). A smaller portion of the teachers held a bachelor’s in special education (*n* = 23, 16.0%) or a postgraduate degree (*n* = 13, 9.0%). The participants were highly experienced, with more than half of the teachers having over 15 years of professional experience (*n* = 76, 52.8%). Regarding their knowledge and training in SEL, a large majority of the teachers had not received any relevant courses (*n* = 120, 83.3%). Despite this lack of formal training, most teachers expressed a desire to attend SEL courses (*n* = 118, 81.9%). When asked about their current knowledge level, the vast majority reported having either basic (*n* = 85, 59.0%) or moderate (*n* = 53, 36.8%) knowledge of SEL. Only a small number of teachers felt they had advanced knowledge (*n* = 6, 4.2%). Detailed demographic information is presented in Table 1.

**Table 1:** The demographic characteristics of the participants (*n* = 144).

Demographic variable	Category	No.	Percentage (%)
Gender	Female	91	63.2
	Male	53	36.8
Academic degree	Bachelor’s/higher diploma	108	75.0
	Bachelor’s in special education	23	16.0
	Postgraduate degree	13	9.0
Job title	General education teacher	114	79.2
	Special education teacher	30	20.8
Years of experience	1–5 years	15	10.4
	6–10 years	12	8.3
	11–15 years	41	28.5
	More than 15 years	76	52.8
Received SEL courses	No	120	83.3
	Yes	24	16.7
Desire to attend SEL courses	Yes	118	81.9
	No	26	18.1
Level of knowledge in SEL	Basic knowledge	85	59.0
	Moderate knowledge	53	36.8
	Advanced knowledge	6	4.2

### 2.4 Data analysis

The quantitative data gathered from the questionnaire were analyzed using the statistical software program, SPSS (Statistical Package for the Social Sciences). The quantitative data gathered from the questionnaire were analyzed using the statistical software program, SPSS (Statistical Package for the Social Sciences). Descriptive statistics, including frequencies and percentages, summarized the demographic profile, while means and standard deviations were used to evaluate participants’ perceptions, practices, and the ranking of perceived challenges. Inferential statistics were employed to identify significant differences at the *α* ≥ 0.05 level: independent-samples *t*-tests compared results by gender and job title, while one-way ANOVA assessed differences across years of experience and educational attainment. Finally, qualitative insights gathered from the three open-ended questions—regarding SEL definitions, implementation improvements, and teacher-facing obstacles—were processed using thematic analysis to identify recurring patterns.

## 3 Results and discussion

The findings are organized into three sections: quantitative results from statistical analyses, qualitative insights from teachers’ perspectives, and an integrated discussion that combines both to provide a comprehensive understanding of the study’s outcomes.

### 3.1 Quantitative results

#### 3.1.1 Teachers’ perceptions of SEL

Based on a survey of teachers, the data in Table 2 reveals a strong, positive perception of SEL for students with ADHD. The overall mean score of 4.06 (on a scale of 1 to 5) indicates that teachers widely agree that SEL is valuable for this student population.

**Table 2:** Teacher responses on their perceptions of SEL for students with ADHD: means, standard deviations, and ranks.

No.	Statement	Rank	Mean	SD
1	SEL is a framework built around five core competencies: self-awareness, social awareness, self-management, relationship management, and responsible decision-making.	3	4.15	0.70
2	SEL helps students diagnosed with ADHD develop key social skills.	1	4.17	0.69
3	SEL helps students diagnosed with ADHD improve their communication skills.	4	4.14	0.79
4	SEL helps students diagnosed with ADHD identify and understand their emotions.	2	4.16	0.68
5	SEL helps students diagnosed with ADHD improve their ability to understand their own thoughts.	5	4.12	0.68
6	SEL helps students diagnosed with ADHD develop a stronger sense of empathy towards others.	7	4.08	0.70
7	SEL focuses on teaching students diagnosed with ADHD social and emotional skills, such as how to manage their relationships with others.	6	4.09	0.69
8	SEL assists students diagnosed with ADHD in making responsible decisions.	10	3.99	0.79
9	Teachers can teach SEL skills within the classroom to students diagnosed with ADHD.	12	3.84	0.87
10	I believe that teaching SEL improves the academic performance of students diagnosed with ADHD.	9	4.01	0.75
11	I believe that teaching SEL reduces behavioral challenges among students diagnosed with ADHD.	11	3.99	0.78
12	I believe teaching SEL to students diagnosed with ADHD is just as important as teaching them academic skills.	8	4.02	0.81
Overall			4.02	0.59

Specifically, the survey found that teachers most strongly believe that SEL helps students with ADHD develop key social skills (M = 4.17). This high level of agreement is consistent across most aspects of SEL, showing a general consensus on its benefits.

Interestingly, while teachers have a positive overall view, the lowest-rated item was the belief that they can teach SEL skills within the classroom (M = 3.84). This suggests that teachers agree on the value of SEL, but may face challenges when it comes to implementation.

#### 3.1.2 Teachers’ practices in implementing SEL

The data presented in Table 3 indicates that teachers are actively implementing SEL for their students with ADHD, as shown by a high overall mean score of 3.96. The highest-rated practice was fostering positive relationship skills, with a mean score of 4.10. This often involved teaching students how to communicate clearly and work co-operatively with others.

**Table 3:** Teachers’ practices on SEL for students with ADHD: means, standard deviations, and ranks.

No.	Statement	Rank	Mean	SD
1	When I plan lessons, I integrate appropriate SEL components into the lesson.	6	3.91	0.84
2	I assess the SEL needs of students diagnosed with ADHD to provide targeted interventions for each individual.	7	3.86	0.84
3	I support self-management skills in students diagnosed with ADHD. For example, I teach students strategies to cope with feelings of stress and frustration that impact their learning.	4	3.93	0.86
4	I help students develop self-awareness skills. For example, I ask students diagnosed with ADHD to reflect on their academic and personal strengths during learning activities.	5	3.92	0.81
5	I help students diagnosed with ADHD improve their social awareness by teaching them to recognize differences and show empathy towards others.	3	3.98	0.79
6	I help students diagnosed with ADHD develop positive relationship skills. For example, I teach them how to communicate clearly, listen effectively, and collaborate with others.	1	4.10	0.69
7	I support responsible decision-making skills. For example, I involve students diagnosed with ADHD in problem-solving strategies.	2	4.02	0.73
Overall			3.96	0.68

Conversely, the lowest-rated practice was assessing the needs of students with ADHD to provide targeted SEL interventions (M = 3.86). This finding may be partially explained by the fact that many of the study participants were general education teachers, who may not have the specialized training or resources needed to perform such individualized assessments.

#### 3.1.3 Challenges in implementing SEL

Teachers face significant obstacles when implementing SEL for students with ADHD. On a scale where a higher number indicates a more significant challenge, teachers rated the overall difficulty as a 3.99, which is considered highly impactful.

The most critical challenge teachers identified was a scarcity of professional development programs related to SEL, with a mean score of 4.13. This suggests that teachers feel a strong need for more training and specialized knowledge to effectively support their students.

Conversely, while still rated as a major issue, the scarcity of suitable SEL resources was identified as the least-rated challenge, with a mean score of 3.90. This indicates that while resources are a concern, access to proper training is seen as a more urgent need.

Other notable challenges highlighted by teachers include a lack of knowledge about SEL, the heavy workload of teachers, and the prioritization of academic skills over SEL in the classroom. Table 4 provides a detailed breakdown of the challenges reported by teachers, along with their average scores and rankings.

**Table 4:** Teacher-reported challenges in implementing SEL for students with ADHD: means, standard deviations, and ranks.

No.	Statement	Rank	Mean	SD
1	Inadequate knowledge of SEL	2	4.04	0.74
2	Insufficient professional preparation regarding SEL	4	3.97	0.83
3	Scarcity of professional development programs related to SEL	1	4.13	0.77
4	Lack of school support regarding SEL	6	3.94	0.85
5	The heavy workload of teachers of students diagnosed with ADHD.	3	3.99	0.86
6	Lack of suitable resources for teachers on SEL	7	3.90	0.82
7	Prioritizing academic skills over SEL skills	5	3.97	0.79
Overall			3.99	0.63

#### 3.1.4 Factors influencing teacher perceptions and implementation of SEL

Teachers’ perceptions and practices of SEL for students with ADHD were analyzed based on four key variables: gender, job title, years of teaching experience, and academic qualification.

The study found statistically significant differences in both the perceptions and practices of SEL between male and female teachers. Female teachers reported higher mean scores for both perceptions (M = 4.14, SD = 0.55) and practices (M = 4.08, SD = 0.56) compared to their male counterparts (perceptions: M = 3.93, SD = 0.64; practices: M = 3.75, SD = 0.80). An independent-samples *t*-test confirmed these differences were statistically significant for both perceptions (*t* = 2.132, *p* = 0.035) and practices (*t* = 2.644, *p* = 0.010). These findings suggest suggests that female teachers may be more aware of students’ social and emotional needs and are more likely to integrate these considerations into their classroom management and interactions.

In contrast, there was no statistically significant difference in SEL perceptions or practices between general education teachers and special education teachers. An independent-samples *t*-test showed that the mean scores for both groups were similar. The *p*-values for both perceptions (*p* = 0.459) and practices (*p* = 0.834) were greater than the significance level of *α* ≤ 0.05, indicating that the observed differences were not statistically significant. This suggests that both general and special education teachers hold com-parable views and employ similar practices related to SEL. Detailed data can be found in Table 5.

**Table 5:** Differences in SEL perceptions and practices according to teachers’ gender and job titles.

Variable	Group	Subgroup	*N*	Mean	SD	*t*-value	*p*-value
Perceptions	Gender	Male	53	3.93	0.64	2.132	0.035
		Female	91	4.14	0.55		
Practices	Gender	Male	53	3.75	0.80	2.644	0.010
		Female	91	4.08	0.56		
Perceptions	Job title	General Ed. teacher	114	4.04	0.62	0.743	0.459
		Special Ed. teacher	30	4.13	0.49		
Practices	Job title	General Ed. teacher	114	3.97	0.69	0.210	0.834
		Special Ed. teacher	30	3.94	0.65		

As shown in Table 6, a one-way analysis of variance (ANOVA) was used to analyze the influence of teachers’ professional experience and academic degrees on their SEL perceptions and practices. For both variables, the results showed no statistically significant differences. The *p*-values for perceptions and practices were greater than the significance level of *α* ≤ 0.05, meaning that the variations in responses were not statistically significant. This indicates that teachers’ years of experience and academic qualifications did not influence their perceptions or practices of SEL.

**Table 6:** Differences in SEL perceptions and practices according to teachers’ professional experience and academic degrees.

Variable	Group	Source of variance	Sum of squares	*df*	Mean square	*F*-value	*p*-value
Perceptions	Years of experience	Between groups	1.500	3	0.500	1.447	0.232
		Within groups	48.399	140	0.346		
		Total	49.899	143			
Practices	Years of experience	Between groups	0.273	3	0.091	0.194	0.901
		Within groups	65.695	140	0.469		
		Total	65.968	143			
Perceptions	Academic degree	Between groups	0.341	2	0.170	0.485	0.617
		Within groups	49.558	141	0.351		
		Total	49.899	143			
Practices	Academic degree	Between groups	0.243	2	0.121	0.260	0.771
		Within groups	65.726	141	0.466		
		Total	65.968	143			

### 3.2 Qualitative results

#### 3.2.1 Teachers’ perceptions of SEL

Based on an analysis of participant responses, SEL is a continuous process that helps students develop crucial social and emotional abilities. These skills enable students to understand and manage their emotions, build positive relationships, and make responsible choices. SEL also supports students in setting and achieving personal goals, which is crucial for success in school, careers, and life, and for their overall well-being within a healthy community. Participants’ perceptions of SEL emphasized several core components:

- Self-awareness & self-management: participants highlighted the importance of understanding and managing one’s own emotions. For example, participant 66 defined SEL as “the developmental process of self-awareness, self-control, and social skills that are essential for achievement in education, career, and life.”
- Social awareness & relationship skills: the ability to build positive relation-ships and effective communication was a key theme. Participant 102 described SEL as “the ability to understand and empathize with the feelings of others; and the ability to communicate effectively and interact appropriately with others.”
- Responsible decision-making: the ability to make sound choices was also a key theme. Participant 107 mentioned “learning the necessary skills to manage emotions, engage in effective social interaction, make sound decisions, and achieve personal and collective goals.”
- Personal goal setting: some participants noted that SEL helps students set and achieve personal goals. Participant 57 offered a comprehensive definition, stating that SEL is the process through which young people “acquire and apply the knowledge and skills to achieve personal and collective goals.”

Participants’ responses also identified several aspects that extend beyond the core components:

- Environmental aspect: many responses emphasized the need for a supportive learning environment that integrates social and emotional dimensions into education. For example, Participant 18 said, “Providing emotional, social, positive support and love is the essence of humanity.”
- Social interaction and relationships: participants underscored the importance of building positive relationships not just in the classroom, but also with the wider community and families. As Participant 97 said, “SEL encompasses the family, the community, and all individuals.”
- Addressing behavioral challenges: participants mentioned using SEL to con-front and respond to students’ behavioral difficulties, modify undesirable behaviors, and offer support for students with challenges like ADHD. Participant 55 described SEL as “Guiding students with behavioral challenges toward positive conduct.”

#### 3.2.2 Teachers’ practices in implementing SEL

Participants suggested several ways to enhance SEL implementation, primarily organized into four key areas:

- Classroom practices: participants recommended integrating emotional and behavioral skills into academic subjects, using activity-based learning, and applying specific methods like short morning meetings, role-playing, and self-reflection. Participant 135 gave a concrete example, saying that when teaching history, “discussions can revolve around characters’ emotions, the rea-sons behind their actions, and how they handled situations.”
- Teacher development: the importance of providing teachers with resources, training, and professional development was a recurring theme. Participant 139 emphasized that “training courses should be provided by academics or re-searchers in the field.” They also suggested that SEL should be integrated into all teacher preparation curricula and that universities and schools should collabo-rate on professional development.
- Policies and planning: several participants called for a structured and systematic plan for SEL implementation. This includes allocating dedicated time for SEL practices, raising awareness about its importance, and even appointing a specific SEL leader or counselor within the school. Participant 38 suggested “appointing an SEL Leader within the school and ensuring continuous professional development.”
- School environment and community: several participants stressed the need for a safe and supportive school environment and the integration of efforts between the school, family, and community. As participant 38 noted, “The integration of efforts between the school, family, and community enhances individuals’ success in their personal and professional lives.”

#### 3.2.3 Challenges in implementing SEL

Challenges related to SEL fall into four main categories, according to participants.

- Knowledge and training: teachers feel they have limited understanding and insufficient training in SEL principles. They also noted a scarcity of resources.
- Time and workload: a demanding workload and lack of time make it difficult for teachers to dedicate enough time to SEL.
- Curriculum and academic focus: the intense focus on academics often sidelines SEL, preventing it from being a central part of the curriculum.
- Classroom environment and students: teachers report large class sizes, challenging student behaviors, and a wide variety of student backgrounds. They also mentioned a lack of family involvement.

One participant summarized these challenges well, stating that teachers may face “time constraints and curriculum pressure, a lack of training and professional support, dealing with diverse student cultural and social backgrounds, potential resistance from students or parents, the influence of the overall school environment, the risk of teacher emotional exhaustion, and the inherent difficulties in measuring impact and evaluation.”

### 3.3 Integrated discussion

Both quantitative and qualitative data show that teachers have a strong, positive view of SEL’s benefits for students with ADHD. The survey results show a high overall mean score for the perception of SEL, with a particularly strong belief that SEL helps students develop key social skills. This is supported by the qualitative findings, which highlighted SEL as a “continuous process” essential for developing a wide range of social and emotional abilities. Both data sets emphasized core SEL components like self-awareness, self-management, social awareness, and relationship skills, suggesting a shared understanding of what SEL entails. These findings align with Buchanan et al. (2009), who argue that SEL is vital for both academic achievement and long-term life outcomes. This perspective is further supported by Doikou (2024), whose research indicates that special education teachers view SEL as essential for the emotional growth and social integration of students with disabilities. Collectively, these studies reinforce the positive perceptions of SEL observed among teachers in the current study.

However, a notable divergence emerged concerning teachers’ confidence in their own ability to implement SEL. The quantitative findings revealed that the lowest-rated perception was teachers’ belief in their capacity to teach SEL skills (M = 3.84). This finding is critically important as it suggests a clear gap between teachers’ positive views on SEL’s value and their perceived capacity to deliver it effectively. This is further substantiated by the qualitative data, which underscored a lack of knowledge and insufficient training as major challenges, directly impacting teachers’ confidence in their implementation abilities. Consistent with previous studies (Daldal and Tuyan, 2024; Doikou, 2024; Ferreira et al., 2021; Humphries et al., 2018; Yoder, 2022), these findings highlight the critical role of teacher preparation. This corroborates Hassani and Schwab’s (2021) review, which posits that the success of SEL interventions for students with special needs is fundamentally tied to the quality of teacher preparation and training.

While both quantitative and qualitative data confirm that teachers actively implement SEL, they also reveal a key discrepancy in implementation priorities. The quantitative findings showed that the most frequently reported practice was helping students develop positive relationship skills (M = 4.10), while the least common practice was assessing students’ needs to provide targeted interventions (M = 3.86). The qualitative data provided context for this finding. Teachers described using various classroom-based practices, such as morning meetings and role-playing, to foster skills like positive relationships, which aligns with the highest-rated quantitative finding. The instructional practices reported by teachers align with the SEL-supporting strategies outlined by Yoder (2022). This consistency is echoed by Doikou (2024), who found that special education teachers utilize diverse practices to foster social–emotional growth in students with disabilities. Furthermore, the findings reflect the work of Ng and Bull (2018), suggesting that teachers often integrate these SEL practices incidentally into daily classroom routines. However, the qualitative results also highlighted the need for more structured approaches, such as appointing an SEL leader or allocating dedicated time for SEL practices. This suggests that while teachers are implementing SEL in their classrooms, they are doing so without a systematic, individualized approach, which is consistent with the low quantitative score for needs assessment.

Both the quantitative and qualitative results aligned in identifying key obstacles to effective SEL implementation. The most significant challenge identified in the quantitative data was a scarcity of professional development programs (M = 4.13), with a lack of knowledge about SEL also being a top concern. This finding was strongly mirrored in the qualitative results, where participants repeatedly mentioned limited understanding and insufficient training. A lack of time due to heavy workloads and the prioritization of academic skills over SEL were also noted in both data sets, highlighting these as pervasive issues. Interestingly, while the quantitative data ranked the scarcity of resources as the lowest-rated challenge (M = 3.90), the qualitative responses offered a more nuanced view. While resources were mentioned, the emphasis was more on the need for structured and systematic support, such as having a designated SEL leader or school-wide policies. This suggests that the issue is not just a lack of materials but a lack of a clear framework and institutional support to guide SEL efforts.

Existing literature consistently highlights significant barriers to the effective implementation of SEL. Teachers—particularly those supporting students with disabilities—frequently face intense time constraints and the burden of multitasking (Doikou, 2024). Beyond individual workload, systemic factors such as overcrowded classrooms, a rigid focus on academic metrics, and insufficient administrative backing further hinder progress (Daldal and Tuyan, 2024). Additionally, researchers have identified a lack of cultural adaptation in SEL programming, limited resources, and weak synergy between schools and families as critical obstacles (Steed et al., 2022; Humphries et al., 2018; Wider et al., 2025).

The quantitative analysis found a statistically significant difference in both the perceptions and practices of SEL between male and female teachers. Female teachers reported higher mean scores for both perceptions and practices. In contrast, the quantitative analysis found no significant differences in perceptions or practices based on a teacher’s job title, years of experience, or academic qualifications. This indicates that a teacher’s professional background, beyond their gender, does not significantly influence their views or implementation of SEL.

Gender-related disparities in how teachers perceive SEL are often rooted in gender socialization, which shapes professional attitudes toward emotion in the classroom. Research consistently indicates that female teachers demonstrate a higher commitment to SEL than their male counterparts, particularly regarding the value placed on emotional expression and interpersonal relationships in the learning process (Buchanan et al., 2009; Daldal and Tuyan, 2024; Scott Loinaz, 2019).

Crucially, these findings suggest that gender serves as a key explanatory variable for how teachers define the boundaries of their pedagogical roles. The divergence appears to stem from differing perceptions of professional responsibility rather than gaps in knowledge or self-efficacy. This aligns with Brackett et al. (2012), who highlighted that broader variations in emotional intelligence and expression between genders (Brackett et al., 2006) directly influence classroom practices.

The qualitative data, while not directly addressing demographic variables, did reinforce the idea that a teacher’s mindset and a supportive environment are crucial, irrespective of their formal training or experience. This further emphasizes that SEL is seen as a universal component of effective teaching, regardless of a teacher’s background.

## 4 Limitations and recommendations for future research

While this study provides valuable insights, several limitations must be acknowledged. First, the study relied on self-reported data regarding teachers’ perceptions and practices. Without direct classroom observations, the findings represent how teachers perceive their work rather than a direct record of actual classroom behavior. Second, the primary data collection relied on a questionnaire. Although the inclusion of open-ended questions allowed for qualitative detail, the format may lack the interpretive depth of in-depth interviews. Finally, the participants was predominantly female and limited to public elementary schools. Consequently, the findings may not be fully generalizable to male educators, private school contexts, or secondary and higher education levels. These results should be interpreted with caution, and future research should employ more diverse samples to enhance generalizability.

Despite the acknowledged limitations, this study offers several significant contributions to the field: First, by incorporating open-ended questions within the survey instrument, the study moved beyond simple quantitative metrics to capture nuanced qualitative insights, aligning with established mixed-methods frameworks (Teddlie and Tashakkori, 2008). Second, by focusing specifically on public elementary schools, the study provides a deep, contextualized understanding of a critical educational sector that is often the foundation for student development. Third, the predominantly female participants, while a limitation for generalizability, provides a highly accurate representation of the current demographic reality of the elementary teaching workforce, where women often make up the vast majority of practitioners. Finally, the findings offer a “baseline” of self-reported practices that can help school administrators and policymakers identify gaps between perceived teacher needs and actual school resources.

Based on the current findings and the identified limitations, future research should consider the following directions: First, future studies should employ triangulation by combining self-reported surveys with direct classroom observations and semi-structured interviews. This would help bridge the gap between perceived and actual classroom practices. Second, implementing a longitudinal study would allow researchers to track how teacher perceptions and practices evolve over time, particularly in response to new professional development initiatives or policy changes. Finally, future studies should make a concerted effort to recruit male educators to determine if gender influences pedagogical approaches or perceptions of school culture at the elementary level. It should also move beyond demographic data to explore the psychological underpinnings of teacher assessments. A concerted effort to include male educators is essential to determine if gendered perspectives alter the delivery of SEL curricula. Furthermore, investigating the intersection of teacher bias and student assessment is vital; research should determine if stereotypical gender norms—such as the expectation of emotional stoicism in males or innate sociability in females—distort the evaluation of student progress. Understanding these dynamics is key to ensuring that SEL implementation is equitable and objective for all students.

## 5 Conclusion

This study explores teachers’ perceptions, implementation, and challenges related to SEL for students with ADHD. Based on the analysis of both quantitative and qualitative data, this study concludes that there is a notable paradox in the implementation of SEL for students with ADHD. Teachers overwhelmingly recognize the value and importance of SEL, viewing it as a critical framework for helping these students develop essential social, emotional, and decision-making skills. However, a significant gap exists between this positive perception and the practical reality of classroom implementation. This suggests that while teachers are motivated to support their students, they feel ill-equipped with the necessary knowledge and resources to do so effectively. Furthermore, the challenges of a heavy workload and the institutional prioritization of academic skills over SEL emerged as key barriers in both data sets, confirming a systemic issue that extends beyond individual teacher capability.

In summary, while the will to implement SEL for students with ADHD exists among educators, a lack of institutional support—especially in terms of training and resources—is a major obstacle. For SEL to be successfully integrated into the curriculum for students with ADHD, the focus must shift from simply affirming its value to actively equipping teachers with the necessary skills and support. Future efforts should concentrate on providing targeted, high-quality professional development and creating a school environment that values SEL as much as academic instruction. Addressing this gap is essential to ensuring that all students, particularly those with ADHD, receive the social and emotional support they need to succeed academically and personally.

## Data Availability

The raw data supporting the conclusions of this article will be made available by the authors, without undue reservation.

## References

[ref1] Bakola L. N. Drigas A. (2020). Technological development process of emotional intelligence as a therapeutic recovery implement in children with ADHD and ASD comorbidity. Int. J. Online Biomed. Eng. 16, 75–85. doi: 10.3991/ijoe.v16i03.12877

[ref2] Bakola L. Rizos N. Drigas A. (2019). ICTs for emotional and social skills development for children with ADHD and ASD co-existence. Int. J. Emerg. Technol. Learn. 14, 22–131. doi: 10.3991/ijet.v14i05.9430

[ref3] Barkley R. A. (1997). Behavioral inhibition, sustained attention, and executive functions: constructing a unifying theory of ADHD. Psychol. Bull. 121, 65–94. doi: 10.1037/0033-2909.121.1.65, 9000892

[ref4] Barkley R. A. (2016). Managing ADHD in school: the best evidence-based methods for teachers. Eau Claire, Wisconsin, USA: Pesi Publishing & Media.

[ref5] Brackett M. A. Reyes M. R. Rivers S. E. Elbertson N. A. Salovey P. (2012). Assessing teachers’ beliefs about social and emotional learning. J. Psychoeduc. Assess. 30, 219–236. doi: 10.1177/0734282911424879

[ref6] Brackett M. A. Rivers S. E. Shiffman S. Lerner N. Salovey P. (2006). Relating emotional abilities to social functioning: a comparison of self-report and performance measures of emotional intelligence. J. Pers. Soc. Psychol. 91, 780–795. doi: 10.1037/0022-3514.91.4.780, 17014299

[ref7] Buchanan R. Gueldner B. A. Tran O. K. Merrell K. W. (2009). Social and emotional learning in classrooms: a survey of teachers’ knowledge, perceptions, and practices. J. Appl. Sch. Psychol. 25, 187–203. doi: 10.1080/15377900802487078

[ref8] Collaborative for Academic, Social, and Emotional Learning (CASEL). (2020). CASEL’s SEL framework: what are the core competence areas and where are they promoted? [PDF file]. Available online at: https://casel.org/casel-sel-framework-11-2020/ (accessed April 11, 2024)

[ref9001] Collaborative for Academic, Social, and Emotional Learning (CASEL). (2023). Fundamentals of SEL. Available online at: https://casel.org/fundamentals-of-sel/ (accessed on June 5, 2024).

[ref9] Daldal D. Tuyan S. (2024). Turkish EFL teachers’ knowledge, perceptions and practices regarding social and emotional learn ing. Int. J. Educ. Res. Rev. 9, 108–118. doi: 10.24331/ijere.1332202

[ref10] Doikou M. (2024) Promoting social and emotional learning in pupils with disability. Special teachers’ perceptions and practices Int. J. Emot. Educ. 16 154–168. Available online at: https://www.um.edu.mt/library/oar/handle/123456789/120925 (accessed on November 18, 2024).

[ref11] Drigas A. Pergantis P. Chaidi I. Christou A. (2025). The KIDSWELL project: supporting teachers to ensure a learning and inclusive environment for children with ADHD based on the introduction of emerging technologies for the development of skills as an active citizen. World J. Adv. Res. Rev. 25, 1807–1818. doi: 10.30574/wjarr.2025.25.3.0945

[ref12] Durlak J. A. Mahoney J. L. Boyle A. E. (2022). What we know, and what we need to find out about universal, school-based social and emotional learning programs for children and adolescents: a review of meta-analyses and directions for future research. Psychol. Bull. 148, 765–782. doi: 10.1037/bul0000383

[ref13] Durlak J. A. Weissberg R. P. Dymnicki A. B. Taylor R. D. Schellinger K. B. (2011). The impact of enhancing students’ social and emotional learning: a meta-analysis of school-based universal interventions. Child Dev. 82, 405–432. doi: 10.1111/j.1467-8624.2010.01564.x, 21291449

[ref14] Ferreira M. Reis-Jorge J. Batalha S. (2021). Social and emotional learning in preschool education-a qualitative study with preschool teachers. Int. J. Emot. Educ. 13, 51–66. Available online at: https://www.um.edu.mt/library/oar/handle/123456789/76514 (accessed on June 21, 2024)

[ref15] Government of Saudi Arabia. (2016). Vision 2030. Available online at: https://www.vision2030.gov.sa/en (accessed December 12, 2024)

[ref16] Greenberg M. T. (2023). Evidence for social and emotional learning in schools. Learning Policy Institute. Available online at: https://learningpolicyinstitute.org/media/3977/download?inline&file=Evidence_for_SEL_REPORT.pdf (accessed December 05, 2024)

[ref17] Hassani S. Schwab S. (2021). Social-emotional learning interventions for students with special educational needs: a systematic literature review. Front. Educ. 6:808566. doi: 10.3389/feduc.2021.808566

[ref18] Humphries M. L. Williams B. V. May T. (2018). Early childhood teachers' perspectives on social-emotional competence and learning in urban classrooms. J. Appl. Sch. Psychol. 34, 157–179. doi: 10.1080/15377903.2018.1425790, 30713472 PMC6352301

[ref9002] Kaur J. Sharma A. (2022). What do I know about social-emotional learning: A comparative analysis between public and private preschool teachers in Punjab. SAGE Open. 12:21582440221091254. doi: 10.1177/21582440221091254

[ref19] Li S. Tang Y. Zheng Y. (2023). How the home learning environment contributes to children’s social–emotional competence: a moderated mediation model. Front. Psychol. 14:1065978. doi: 10.3389/fpsyg.2023.1065978, 36865364 PMC9971822

[ref20] Ng S. C. Bull R. (2018). Facilitating social emotional learning in kindergarten classrooms: situational factors and teachers’ strategies. Int. J. Early Child. 50, 335–352. doi: 10.1007/s13158-018-0225-9

[ref21] Payton J. Weissberg R.P. Durlak J.A. Dymnicki A.B. Taylor R.D. Schellinger K.B. . (2008). The positive impact of social and emotional learning for kindergarten to eighth-grade students: findings from three scientific reviews. Chicago, IL: Collaborative for Academic, Social, and Emotional Learning. Available online at: https://files.eric.ed.gov/fulltext/ED505370.pdf (accessed on April 05, 2024)

[ref9003] Schonert-Reichl K. A. (2017) Social and emotional learning and teachers The future of children. 137–155. Available online at: http://www.jstor.org/stable/44219025

[ref22] Scott Loinaz E. (2019) Teachers’ perceptions and practice of social and emotional education in Greece, Spain, Sweden and the United Kingdom Int. J. Emot. Educ. 11 31–48. Available online at: https://www.um.edu.mt/library/oar//handle/123456789/42655 (accessed on January 3, 2025).

[ref23] Steed E. A. Shapland D. Leech N. (2022). Early childhood teachers’ perceptions of the effectiveness of their elementary school’s approach to social emotional learning: a mixed methods study. Early Child. Educ. J. 50, 1121–1132. doi: 10.1007/s10643-021-01248-4

[ref24] Teddlie C. Tashakkori A. (2008). Foundations of mixed methods research: integrating quantitative and qualitative approaches in the social and behavioral sciences. Thousand Oaks: Sage publications.

[ref25] Wider W. Shareefa M. Moosa V. Ng M. L. Isa A. M. B. M. Fauzi M. A. . (2025). Mapping the terrain of social–emotional learning: a bibliometric study on its past, present, and future. Sch. Ment. Health 17, 1097–1112. doi: 10.1007/s12310-025-09781-y

[ref26] Yoder N. (2022). Self-Assessing Social and Emotional Instruction and Competencies: A Tool for Teachers. Center on Great Teachers and Leaders at the American Institutes for Research (AIR). Arlington, VA. Available online at: https://www.air.org/sites/default/files/2022-12/GTL-Educator-Self-Assessment-Refreshed-2014-508.pdf (accessed on 08 April 2024).

