# Correction: Social–Emotional Learning for students with ADHD: investigating teacher perspectives and practices

**DOI:** 10.3389/fpsyg.2026.1906857

**Published:** 2026-07-20

**Authors:** Ghram A. S. Kushi, Keetam D. F. Alkahtani

**Affiliations:** 1 Doctoral Program at the Department of Special Education, College of Education, King Saud University, Riyadh, Saudi Arabia; 2 Department of Special Education, College of Education, King Saud University, Riyadh, Saudi Arabia

**Keywords:** Attention Deficit/Hyperactivity Disorder (ADHD), mixed-methods, self-assessment, Social–Emotional Learning (SEL), teachers, inclusive education, holistic student development

Affiliation “Doctoral Program at the Department of Special Education, College of Education, King Saud University, Riyadh, Saudi Arabia” was omitted for author Ghram A. S. Kushi. This affiliation has now been added for author Ghram A. S. Kushi.

Affiliation “Department of Special Education, College of Education, King Saud University, Riyadh, Saudi Arabia” was erroneously assigned to author Ghram A. S. Kushi. This affiliation has now been removed for author Ghram A. S. Kushi.

An incorrect **Funding** statement was provided. The correct **Funding** statement reads:

The author(s) declared that financial support was received for this work and/or its publication. The authors thank the Ongoing Research Funding program (ORF-2026-1328), King Saud University, Riyadh, Saudi Arabia.

The original version of this article has been updated.

